# *Melicope stonei*, section Pelea (Rutaceae), a new species from Kaua‘i, Hawaiian Islands: with notes on its distribution, ecology, conservation status, and phylogenetic placement

**DOI:** 10.3897/phytokeys.83.13442

**Published:** 2017-08-03

**Authors:** Kenneth R. Wood, Marc S. Appelhans, Warren L. Wagner

**Affiliations:** 1 National Tropical Botanical Garden, 3530 Papalina Road, Kalāheo, HI 96741, USA; 2 Department of Systematics, Biodiversity and Evolution of Plants, Albrecht-von-Haller Institute of Plant Sciences, University of Göttingen, Untere Karspüle 2, 37073 Göttingen, Germany; 3 Department of Botany, Smithsonian Institution, PO Box 37012, Washington, DC 20013-7012, USA

**Keywords:** Rutaceae, *Melicope*, *M.* section *Pelea*, new species, conservation, Hawaiian Islands, Kaua‘i, Critically Endangered

## Abstract

*Melicope stonei* K.R. Wood, Appelhans & W.L. Wagner (section Pelea, Rutaceae), a new endemic tree species from Kaua‘i, Hawaiian Islands, is described and illustrated with notes on its distribution, ecology, conservation status, and phylogenetic placement. The new species differs from its Hawaiian congeners by its unique combination of distinct carpels and ramiflorous inflorescences arising on stems below the leaves; plants monoecious; leaf blades (5–)8–30 × (4–)6–11 cm, with abaxial surface densely tomentose, especially along midribs; and very long petioles of up to 9 cm. Since its discovery in 1988, 94 individuals have been documented and are confined to a 1.5 km^2^ region of unique high canopy mesic forest. *Melicope stonei* represents a new Critically Endangered (CR) single island endemic species on Kaua‘i.

## Introduction

The genus *Melicope* J.R. Forst. & G. Forst. consists of ca. 235 species of shrubs and trees with a distribution that ranges from the Malagasy and Indo-Himalayan regions in the east to the Hawaiian and Marquesan Islands in the west and from Nepal, southern China, Taiwan and the Japanese Ogasawara Islands in the north to New Zealand and Australia in the south (Hartley 2001, Appelhans et al. 2014a, Wood et al. 2016a). With the inclusion of *Melicope stonei* K.R. Wood, Appelhans & W.L. Wagner, the total number of recognized *Melicope* reported for the Hawaiian Islands totals 50 endemic species, making *Melicope* the most species-rich radiation of woody plants throughout the archipelago (Hillebrand 1888, Hartley and Stone 1989, Wagner et al. 1999, Wood et al. 2016a). Molecular phylogenetic studies indicate that the Hawaiian species arose from a single introduction, and that the Hawaiian genus *Platydesma* H. Mann is nested within Melicope section Pelea (A. Gray) Hook. f. as sister to the Hawaiian species of *Melicope* (Harbaugh et al. 2009, Appelhans et al. 2014b, c). To preserve the monophyly of Melicope sect. Pelea, *Platydesma* must be merged with *Melicope* and when those new combinations are validly published, Hawaiian *Melicope* will then be increased by an additional four species.

In the most current systematic revision by Hartley (2001) *Melicope* was subdivided into four sections: *Lepta* (Lour.) T.G. Hartley; *Melicope*; *Pelea*; and Vitiflorae T.G. Hartley. Only sect. Lepta proved to be a monophyletic group in a recent molecular study (Appelhans et al. 2014b). All Hawaiian species of Melicope are members of sect. Pelea, which consists of 86 species almost exclusively restricted to Melanesia and the Pacific region. *Pelea* previously was recognized at the genus rank with the Hawaiian species subdivided into four sections in the revision by Stone (1969): *Apocarpa* B.C. Stone; *Cubicarpa* B.C. Stone; *Megacarpa* B.C. Stone; and *Pelea* (Wagner et al. 1999). The classification needs to be revised since the sectional classification of Hartley (2001) would require the Hawaiian groups to be treated as subsections if it is appropriate to continue recognizing them at all. Within these four Hawaiian groups only the Hawaiian *Pelea* group proved to be monophyletic (Appelhans et al. 2014c).

## Methods

All measurements were taken from dried herbarium specimens and field notes and are presented in the descriptions as follows: length × width, followed by units of measurements (mm or cm). The authors have examined all specimens cited. The extent of occurrence and area of occupancy for *Melicope stonei* was calculated by using ArcMap 10.2 in relation to coordinates recorded while collecting herbarium specimens or making field observations.

## Taxonomic treatment

### Melicope stonei

Taxon classificationPlantaeSapindalesRutaceae

K.R.Wood, Appelhans & W.L.Wagner sp. nov.

urn:lsid:ipni.org:names:77164681-1

Figs 1 , 2A–F

#### Diagnosis.

Differs from Hawaiian congeners by its combination of distinct carpels and ramiflorous inflorescence; plants monoecious; leaf blades (5–)8–30 × (4–)6–11 cm, with abaxial surface tomentose, especially along midribs; and very long petioles of up to 9 cm.

#### Type.

United States of America. Hawaiian Islands, Kaua‘i: Waimea District, Mākaha Valley, *Metrosideros-Acacia* montane mesic forest, 22°7'1.8258"N; 159°40'45.534"W, 997 m elev., 24 Jan 2016, *K. R. Wood & Kahekili Lee 16727* (holotype: PTBG-073080!; isotypes: BISH!, K!, MO!, NY!, UC!, US!).

**Figure 1.**
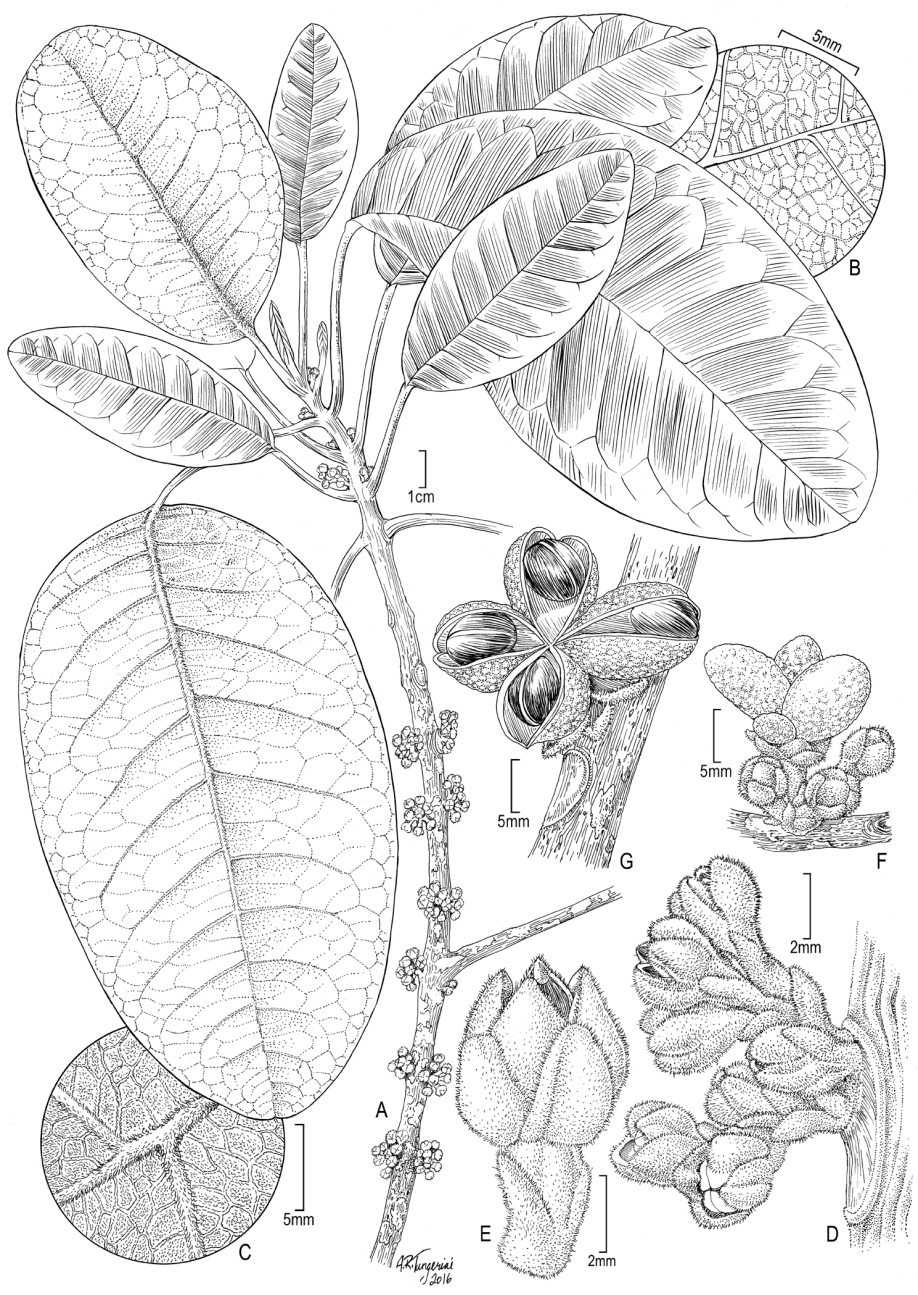
*Melicope stonei* K.R. Wood, Appelhans & W.L. Wagner. **A** Flowering branch **B** Adaxial leaf surface near margin toward apex **C** Abaxial leaf surface near margin toward apex **D** Ramiflorous inflorescence arising below leaves on stem **E** Female flower, lateral view **F** Immature fruit and flowers **G** Dehisced fruit, showing seeds. **A-C** from *Wagner & Wood 6891* (US) **D** from *Wood 8431* (US) **E** from *Wood 15101* (PTBG) **F** from *Wood & Lee 16729* (photo) **G** from *Lorence et al. 6454* (photo) (Illustration by Alice Tangerini).

#### Description.

***Trees*** (3–)5–12 m tall, trunks up to 25 cm diameter, bark smooth, mottled gray to light brown, new growth and young branchlets tomentose, yellow-tan, glabrate in age. ***Leaves*** opposite, unifoliolate, coriaceous, the blade ovate, oblong-ovate, oblong-elliptic, or oblong-lanceolate, (5–)8–30 × (4–)6–11 cm, margin entire, base rounded, apex obtuse to acute, occasionally rounded, secondary veins 7–15 pairs, connected by an arched vein 5–20 mm from margin, higher order venation reticulate, adaxial surface glabrous, abaxial surface densely tomentose, yellow-tan, rarely glabrate, with midrib and secondary veins densely tomentose, interspersed villous, the midrib sometimes also long-villous or woolly along the sides; petiole shallowly canaliculate, (2.5–)3.5–9.0 cm long, puberulent to tomentose. ***Inflorescences*** in axillary and ramiflorous, fasciculate cymes to 22 mm long, peduncles 1–5(–10) mm long, short-villous, pedicles 1–4 mm, short-villous, bracteoles deltoid-ovate, 1–2 mm long, densely short-villous. ***Flowers*** male or female, plants monoecious, 3–5(–7), sepals deltoid-ovate, naviculate, 2.5–3.0 × 1.5–2.0 mm, densely short-villous externally, glabrous internally, tip acute, persistent in fruit; petals whitish green, ovate, tip acute, slightly thickened and hooked inward, 3.5 × 2.5 mm, densely short-villous externally, glabrous internally; stamens 8, filaments glabrous, papillose on distal half, antisepalous ones ca. 4 mm long in male flowers (1.5 mm in female flowers), antipetalous ones 3 mm long (1.5 mm in female flowers), anthers ca. 1 mm long, all with pollen in male flowers, (0.6–0.7 mm long in female, apparently sterile); nectary disk glabrous; ovary glabrous; gynoecium 1 × 2 mm, style including stigma 1 mm long in male flowers (1.5 mm in female), glabrous, stigmas capitate, 4-lobed, papillose, 0.5 mm wide. ***Capsules*** medium to dark green when fresh, irregularly pusticulate, 5–9 × 15–21 mm, of 4 distinct follicles, slightly ascending, occasionally 1 or more abortive, exocarp glabrous, glandular punctate, endocarp glabrous. ***Seeds*** 1–2 per carpel, ovoid, 6–8 mm long.

#### Phenology.

*Melicope stonei* has been observed with flower buds in January, May, and September, and with both flower and fruit during January, February, and July.

#### Etymology.

We are pleased to name *Melicope stonei* in honor of Benjamin Clemens Masterman Stone, British-American botanist, born in Shanghai, China in1933 and passed in 1994 while working at the Philippine National Museum on the Flora of the Philippines Project. He contributed over 300 publications to science during his career, including taxonomic monographs of Hawaiian *Pelea* (Stone 1969) and *Platydesma* (Stone 1962). For all his contributions, especially his keen insights into Hawaiian *Melicope*, we gratefully extend him due recognition.

#### Specimens examined.

**United States. Hawaiian Islands, Kaua‘i**: Waimea District, Ku‘ia, upper headwater gulch, 1027 m elev., 03 Sep 2015, *K. R. Wood, S. Perlman, S. Walsh, M. Query 16579* (PTBG); Miloli‘i ridge road, Mahanaloa, 933 m elev., 06 Nov 2008, *C. Trauernicht & N. Tangalin 617* (PTBG); Mahanaloa, 1055 m elev., 21 Oct 2015, *S. Walsh & A. Williams 136* (PTBG); Pa‘aiki-Mahanaloa flats, 1036 m elev., 07 Oct 2001, *K. R. Wood 9128* (BISH, PTBG); Pa‘aiki-Mahanaloa flats, 991 m elev., 04 Mar 2001, *K. R. Wood & M. LeGrande 8879* (PTBG); Pa‘aiki-Mahanaloa flats, 1000 m elev., 14 Dec 1994, *K. R. Wood 3840* (PTBG, US); Pa‘aiki-Mahanaloa flats, 1000 m elev., 24 Feb 1999, *K. R. Wood 7696* (PTBG); Pa‘aiki-Mahanaloa flats, 1000 m elev., 24 Feb 1999, *K. R. Wood & R. Aguraiuja 7697* (BISH, CANB, MO, NY, PTBG, US); Pa‘aiki-Mahanaloa flats, 1015 m elev., 06 May 2000, *K. R. Wood 8432* (BISH, NY, PTBG, US); Pa‘aiki-Mahanaloa flats, 1015 m elev., 06 May 2000, *K. R. Wood 8433* (PTBG, US); Mākaha Ridge Road, down .8 mi to 988 m elev., north side of road, just inside forest, 970 m elev., 7 Feb 1988, *D. Lorence, G. Lorence & E. Crum 5779* (PTBG); loc. cit., 970 m elev., 2 Jan 1989; *D. Lorence, T. Flynn & Smith 6316* (PTBG); loc. cit., 970 m elev., 1 Mar 1989, *T. Flynn & Decker 3280* (AD, BISH, F, MO, MU, PTBG, US); loc. cit., 970 m elev., 31 May 1990, *D. Lorence et al. 6449* (PTBG); loc. cit., 920 m elev., 25 Feb 2000, *W. Wagner & K. R. Wood 6891* (PTBG); south side of Mākaha road, 988 m elev., 06 May 2000, *K. R. Wood 8430* (PTBG); loc. cit., 988 m elev., 06 May 2000, *K. R. Wood 8431* (PTBG, US); loc. cit., 991 m elev., 21 Jul 2012, *K. R. Wood & T. Bierly 15104.02* (PTBG); loc. cit., 997 m elev., 24 Jan 2016, *K. R. Wood & K. Lee 16726* (BISH, PTBG); loc. cit., 997 m elev., 24 Jan 2016, *K. R. Wood, K. Lee 16727* (PTBG); loc. cit., 997 m elev., Jan 2016, *K. R. Wood, S. Perlman & R. Aguraiuja 16729* (BISH, PTBG, US); loc. cit., 997 m elev., *K. R. Wood & K. Lee 16728* (BISH, K, MO, NY, PTBG, UC, US); loc. cit., 997 m elev., 28 Jan 2016, *K. R. Wood, S. Perlman & R. Aguraiuja 16730* (PTBG, US); loc. cit., 997 m elev., 28 Jan 2016, *K. R. Wood, S. Perlman & R. Aguraiuja 16731* (BISH, PTBG, US); loc. cit., 997 m elev., 28 Jan 2016, *K. R. Wood, S. Perlman & R. Aguraiuja 16732* (BISH, PTBG, UC, US); Mākaha, forests around dividing ridge between upper north and south fork, 1037 m elev., 18 Feb 2016, *K. R. Wood & S. Perlman 16741* (PTBG); Nu‘ololo, north facing slopes above northern branch, 1097 m elev., 18 Jul 2012, *K. R. Wood, M. Query & M. Kirkpatrick 15101* (BISH, K, MBK, MO, NY, P, PTBG, UC, US); Nu‘ololo, north of trail, headwaters of central Nu‘ololo stream, 1061 m, 3 Oct 2012, *K. R. Wood, M. Kirkpatrick & M. Query 15267* (BISH, PTBG, US); Nu‘ololo, 1052 m elev., 02 Jan 2013, *K. R. Wood & M. Kirkpatrick 15319* (BISH, PTBG); Nu‘ololo, 1049 m elev., 02 Jan 2013, *K. R. Wood & M. Kirkpatrick 15320* (BISH, NY, PTBG, UC, US); Nu‘ololo, 1085 m elev., *K. R. Wood, M. Kirkpatrick & S. Perlman 15560* (PTBG); Nu‘ololo, 1027 m elev., Jul 2013, *K. R. Wood, M. Kirkpatrick & S. Perlman 15562* (PTBG); Nu‘ololo, 1052 m elev., 12 Sep 2013, *K. R. Wood & W. Kishida 15667* (PTBG); Nu‘ololo, 1036 m elev., 12 Sep 2013, *K. R. Wood & W. Kishida 15668* (PTBG); Nu‘ololo, 1061 m elev., 28 Sep 2013, *K. R. Wood & M. Query 15671* (PTBG); Nu‘ololo, 1073 m elev., 22 Jul 2014, *K. R. Wood, M. Kirkpatrick, S. Perlman & R. Aguraiuja 16001* (PTBG); loc. cit., 1073 m elev., 22 Jul 2014, *K. R. Wood, M. Kirkpatrick, S. Perlman & R. Aguraiuja 16002* (PTBG).

**Distribution, ecology, and threats.** *Melicope stonei* is endemic to Kaua‘i, oldest of the high Hawaiian Islands at 5.1 million years, with an area of ca. 1430 km^2^ (Sakai et al. 2002), and maximum elevation of 1598 m at the summit of Kawaikini. Floristically, Kaua‘i has the phenomenal distinction of having the highest level of plant diversity of all the Hawaiian Islands, which includes 249 single island endemic (SIE) taxa, 232 of which are flowering plants, and the remaining 17 being unique fern taxa (Palmer 2003, Wagner et al. 2012, Vernon and Ranker 2013, Ranker 2016, Wood et al. 2016b).

Earliest known collections of *Melicope stonei* were made by David Lorence and Timothy Flynn (National Tropical Botanical Garden) as far back as February of 1988 within the forests of Mākaha Valley, Kaua‘i. Over the past 29 years, 94 trees have been mapped by local botanists, with colonies extending into several valleys to the north of Mākaha, namely Ku‘ia, Miloli‘i, Mahanaloa, Nu‘ololo, and Pa‘aiki (Figure 3). *Melicope stonei* has a very narrow elevational range of 988 to 1097 m and a precariously small extent of occurrence of 1.5 km^2^. Rich forest habitats still flourish in adjacent lower and higher elevational regions, yet it is evident that *M. stonei* prefers very tall (15–20 m) old growth *Metrosideros polymorpha* Gaudich. (Myrtaceae)- *Acacia koa* A. Gray (Fabaceae) mixed mesic forest with the occasional co-dominant *Alphitonia ponderosa* Hillebr. (Rhamnaceae). This plant community type only occurs on Kaua‘i and is exceedingly limited with some of the finest examples occurring around the forested flats of Kōke‘e State Park above Pa‘aiki, Mahanaloa, and Nu‘ololo. In these forests, *M. stonei* reaches heights of 10–12 m and is associated with a wide diversity of other tall understory trees such as *Bobea brevipes* A. Gray (Rubiaceae), *Cryptocarya mannii* Hillebr. (Lauraceae), *Dodonaea viscosa* Jacq. (Sapindaceae), *Kadua affinis* DC. (Rubiaceae), *Melicope barbigera* A. Gray, *Myrsine lanaiensis* Hillebr. (Primulaceae), *Nestegis sandwicensis* (A. Gray) O. Deg., I. Deg. & L.A.S. Johnson (Oleaceae), *Planchonella sandwicensis* (A. Gray) Pierre (Sapotaceae), *Polyscias kavaiensis* (H. Mann) Lowry & G.M. Plunkett and *P. waimeae* (Wawra) Lowry & G.M. Plunkett (Araliaceae), *Psychotria mariniana* (Cham. & Schltdl.) Fosberg (Rubiaceae), *Santalum pyrularium* Hook. & Arn. (Santalaceae), *Syzygium sandwicensis* (A. Gray) Nied. (Myrtaceae), *Xylosma hawaiiense* Seem. (Salicaceae), and *Zanthoxylum dipetalum* H. Mann (Rutaceae).

Interspersed below this community’s canopy are rich assemblages of medium statured trees that include Antidesma platyphylla H. Mann var. hillebrandii Pax & Hoffm. (Phyllanthaceae), *Chrysodracon aurea* (H. Mann) P.-L. Lu & Morden (Asparagaceae), *Claoxylon sandwicense* Müll. Arg. (Euphorbiaceae), *Coprosma foliosa* A. Gray and *C. waimeae* Wawra (Rubiaceae), *Cyanea leptostegia* A. Gray (Campanulaceae), *Elaeocarpus bifidus* Hook. & Arn. (Elaeocarpaceae), *Labordia helleri* Sherff (Loganiaceae), *Myrsine alyxifolia* Hosaka, *Pittosporum kauaiense* Hillebr. (Pittosporaceae), *Platydesma rostrata* Hillebr. and *P. spathulata* (Rutaceae), *Psychotria greenwelliae* Fosberg, *Psydrax odorata* (G. Forst.) A.C. Sm. & S.P. Darwin (both Rubiaceae), *Sophora chrysophylla* (Salisb.) Seem. (Fabaceae), *Streblus pendulinus* (Endl.) F. Muell. (Moraceae), and *Wikstroemia furcata* (Hillebr.) Rock (Thymelaeaceae).

Common shrubs and smaller tree components are *Leptecophylla tameiameiae* (Cham. & Schlecht.) C.M. Weiller (Epacridaceae), *Lysimachia kalalauensis* Skottsb. (Primulaceae), *Melicope anisata* (H. Mann) T.G. Hartley & B.C. Stone, *M. feddei* (H. Lév.) T.G. Hartley & B.C. Stone, *M. ovata* (H. St. John & E.P. Hume) T.G. Hartley & B.C. Stone, and *M. peduncularis* (H. Lév.) T.G. Hartley & B.C. Stone.

Associated sedges (Cyperaceae) include *Carex meyenii* Nees, *C. wahuensis* C.A. Mey., and *Gahnia beecheyi* H. Mann, grasses (Poaceae) are *Eragrostis grandis* Hillebr., *E. variabilis* (Gaudich.) Steud., and *Panicum nephelophilum* Gaudich., and additional associated vegetation includes the herbaceous *Dianella sandwicensis* Hook. & Arn. (Xanthorrhoeaceae), and vines of *Alyxia stellata* (J.R. & G. Forst.) Roem. & Schult. (Apocynaceae), and *Smilax melastomifolia* Sm. (Smilacaceae).

Common to occasional pteridophytes of this habitat are *Asplenium normale* D. Don (Aspleniaceae) *Adenophorus tamariscinus* (Kaulf.) Hook. & Grev. and *A. tripinnatifidus* Gaudich. (Polypodiaceae), *Cibotium nealiae* O. Deg. (Cibotiaceae), *Coniogramme pilosa* (Brack.) Hieron. (Pteridaceae), *Diplazium sandwicensis* (A. DC.) Fosberg (Athyriaceae), *Doodia kunthiana* Gaudich. (Blechnaceae), *Dryopteris glabra* (Brack.) Kuntze, and *D. unidentata* (Hook. & Arn.) C. Chr. (Dryopteridaceae), *Elaphoglossum paleaceum* (Hook. & Grev.) Sledge (Dryopteridaceae), *Microlepia strigosa* (Thunb.) C. Presl (Dennstaedtiaceae), and *Odontosoria chinensis* (L.) J. Sm. (Lindsaeaceae).

Threats to *Melicope stonei* include habitat degradation by introduced pigs (*Sus scrofa* L.) and mule deer (*Odocoileus hemionus* Rafinesque), predation of seeds by rats (*Rattus rattus* L. and *R. exulans* Peale), environmental events such as hurricanes, fire (mostly by humans), and competition with invasive non-native plant species, including *Adiantum hispidulum* Sw. (Pteridaceae), *Blechnum appendiculatum* Willd. (Blechnaceae), *Corynocarpus laevigatus* J.R. Forst. & G. Forst. (Corynocarpaceae), *Erigeron karvinskianus* DC. (Asteraceae), *Grevillea robusta* A. Cunn. ex R. Br. (Proteaceae), *Hedychium gardnerianum* Ker Gawl. (Zingiberaceae), *Kalanchoe pinnata* (Lam.) Pres. (Crassulaceae), *Lantana camara* L. (Verbenaceae), *Lophospermum confertus* (R. Br.) P.G. Wilson & J.T. Waterhouse (Myrtaceae), *Morella faya* (Ait.) Wilbur (Myricaceae), *Psidium cattleianum* Sabine (Myrtaceae), *Rubus argutus* Link and *R. rosifolius* Sm. (Rosaceae), *Setaria parviflora* (Poir.) Kerguélen (Poaceae), and *Sphaeropteris cooperi* (Hook. ex F. Muell.) R.M. Tryon (Cyatheaceae) all of which possess the ability to spread rapidly and cover effectively large areas (Smith 1985).

**Conservation status.** *IUCN Red List Category.* When evaluated using the World Conservation Union (IUCN) criteria for endangerment (IUCN 2001), *Melicope stonei* falls into the Critically Endangered (CR) category, which designates this species as facing a very high risk of extinction in the wild. Our formal evaluation can be summarized by the following IUCN hierarchical alphanumeric ranking system of criteria and subcriteria: CR B1ab(i,ii,iii,iv,v)+2ab(i,ii,iii,iv,v); C2a(i); which reflects a severely limited Extent of Occurrence (EOO) and Area of Occupancy (AOO) of less than 1.5 km^2^ and a wild population of less than 100 individuals with all facing a continuing decline in their area of extent and quality of habitat (see Distribution, ecology, and threats). Seeds from several individuals of *M. stonei* have been collected and submitted to the NTBG Horticultural Department for cultivation.

**Morphology, related taxa, and phylogenetic placement.** *Melicope stonei* stands apart from all other described Hawaiian *Melicope* species by its combination of being large monoecious trees up to 12 m tall with distinct carpels and short-villous ramiflorous inflorescences. There are three other Hawaiian species that usually have ramiflorous cymes arising on stems below the leaves, namely *M. clusiifolia* (A. Gray) T.G. Hartley & B.C. Stone from Kaua‘i, O‘ahu, Moloka‘i, Lana‘i, Maui, and Hawai‘i, *M. haleakalae* (B.C. Stone) T.G. Hartley & B.C. Stone from Maui, and *M. waialealae* (Wawra) T.G. Hartley & B.C. Stone from Kaua‘i, yet they differ from *M. stonei* in having leaves in whorls of 4–8 with abaxial surface glabrous or with some hairs on midrib or sometimes loosely villous throughout the surface, and having carpels connate at their base or nearly throughout their length in fruit (as compared to *M. stonei* having leaves opposite with abaxial surface densely tomentose and with carpels distinct in fruit). Other Hawaiian species that may occasionally have the cymes arising below the leaves include *M. ovata* from Kaua‘i, *M. pseudoanisata* (Rock) T.G. Hartley & B.C. Stone, from Maui and Hawai‘i, and *M. quadrangularis* (H. St. John & E.P. Hume) T.G. Hartley & B.C. Stone, also from Kaua‘i. The latter two differ with carpels connate and leaves glabrous or with some hairs on midrib (Wagner et al. 1999), and the former, *M. ovata*, lacks the short-villous peduncles, pedicels, sepals, and petals found on *M. stonei*. Morphologically, these species have little else in common with *M. stonei*.

**Figure 2.**
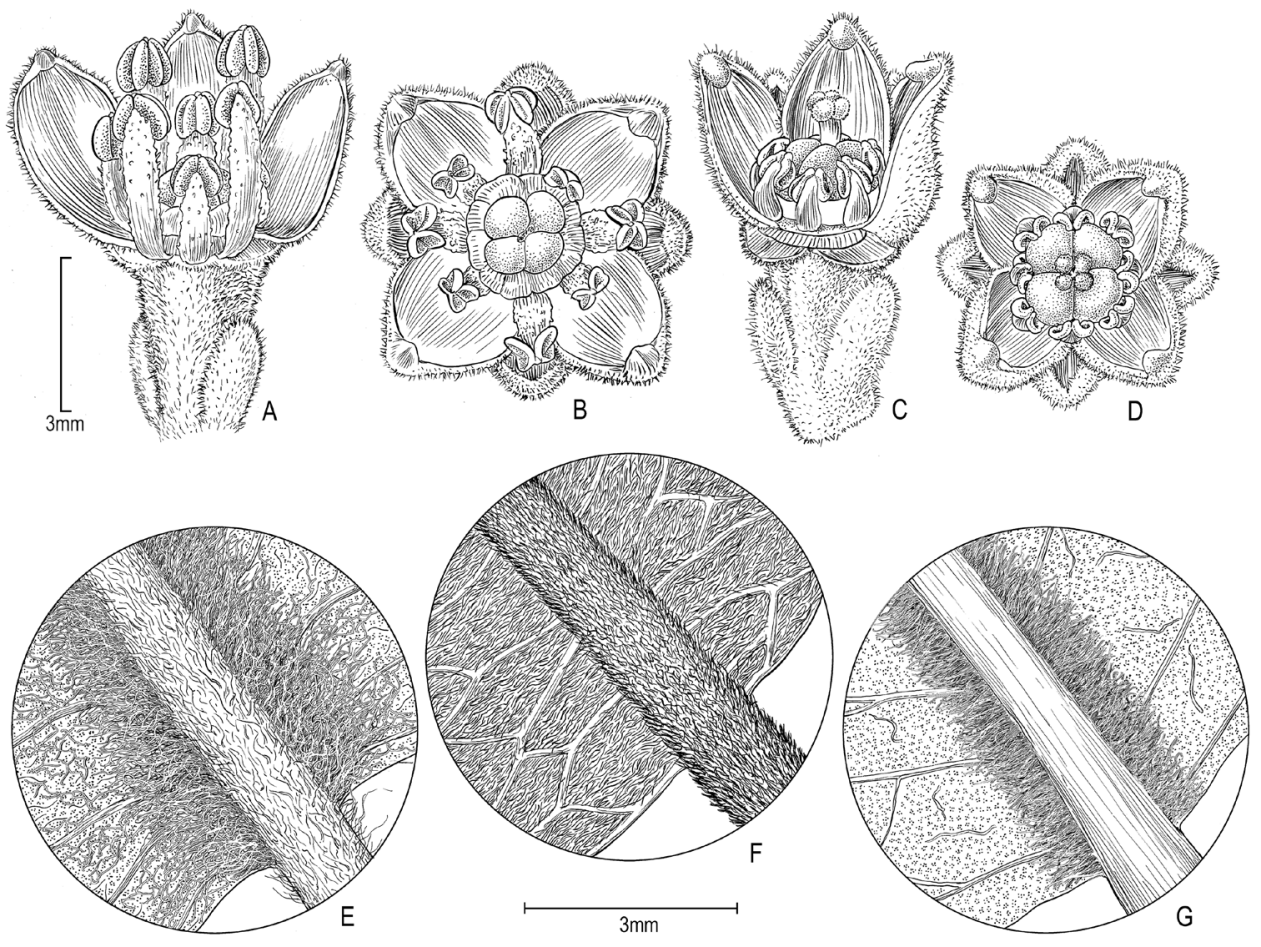
*Melicope stonei* K.R. Wood, Appelhans & W.L. Wagner. **A** Male flower, lateral view with sepals and petals cut away to show antisepalous and antipetalous stamens **B** Male flower, top view **C** Female flower, lateral view with sepals and petals cut away to show staminodes and pistil **D** Female flower, top view **E–F** abaxial leaf surface along midvein of *M. stonei* showing variability of leaf vestiture **G** *Melicope barbigera* A. Gray abaxial leaf surface along midvein **A–D** from *Wood 15101* (PTBG) **E** from *Wagner & Wood 6891* (US) **F** from *Wood 8432* (US) **G** from *Wagner & Wood 6896* (US) (Illustration by Alice Tangerini).

In habit *Melicope stonei* appears most similar to trees of *M. barbigera* from Kaua‘i and *M. knudsenii* (Hillebr.) T.G. Hartley & B.C. Stone from Kaua‘i and Maui, both of which can reach heights of 12 m. *Melicope barbigera* differs from *M. stonei* in having new growth grayish appressed puberulent; slightly folded leaves with waxy scurf and commonly having abaxial leaf surface densely long-villous along each side of midrib (Fig. 2G); cymes axillary, not ramiflorous; peduncles 20–25 mm long; nectary disk and ovary puberulent (as compared to *M. stonei* with new growth tomentose, yellow-tan; leaves neither folded nor having a waxy scurf, rarely with abaxial surface densely long-villous along each side of midrib (Fig. 2E–F); cymes axillary and ramiflorous; peduncles 1–5(–10) mm long; with nectary disk and ovary glabrous). *Melicope knudsenii* differs in having leaf bases weakly cordate; flowers perfect or unisexual, (3–)20–200; cymes axillary, not ramiflorous; and peduncles (10–)30–60 mm long; (as compared to *M. stonei* with leaf bases rounded; flowers male or female, plants monoecious, 3–5(–7); cymes axillary and ramiflorous; and peduncles 1–5(–10) mm long) (Wagner et al. 1999).

Four specimens of *Melicope stonei* (*Wagner & Wood 6891; Wood 7696; Wood & Aguraiuja 7697; Wood, Query & Kirkpatrick 15101*) have been included in phylogenetic analyses of Hawaiian *Melicope* (Harbaugh et al. 2009; Appelhans et al. 2014b,c) and they all represent paratypes of this new species. The specimens were labeled as *M. knudsenii* in these studies according to their original conferred determination. Phylogenetic analyses showed that the former section Apocarpa is paraphyletic with respect to the former section Pelea (unpublished results) and that *M. stonei* forms a clade with the “*Apocarpa*” species *M. adscendens* and *M. ovata* (Fig. 4). The monophyly of this clade is supported by posterior probability (1.00pp) and bootstrap (98bs) values and the three species, of which three to four specimens have been sampled, were resolved as monophyletic entities (Appelhans et al. 2014c; Fig. 4).

*Melicope adscendens* (H. St. John & E.P. Hume) T.G. Hartley & B.C. Stone, from Maui, and the previously mentioned *M. ovata* from Kaua‘i, which are the closest relatives of *M. stonei*, (Appelhans et al. 2014c; Fig. 4) do not have clear morphological similarities with the new species. While *M. stonei* is a tall tree, *M. adscendens* is a sprawling shrub and *M. ovata* is a shrub or small tree with sprawling branches. *Melicope adscendens* has considerably smaller leaves 1.5–6.5 × 1–4 cm and does not have a densely tomentose abaxial leaf surface, being minutely puberulent, becoming glabrate (as compared to *M. stonei* with leaves (5–)8–30 × (4–)6–11 cm with densely tomentose abaxial leaf surface). *Melicope ovata* has sepals glabrous or sparsely minutely ciliate externally, and petals glabrous externally (Wagner et al. 1999), (as compared to *M. stonei* with sepals and petals densely short-villous externally).

**Figure 3.**
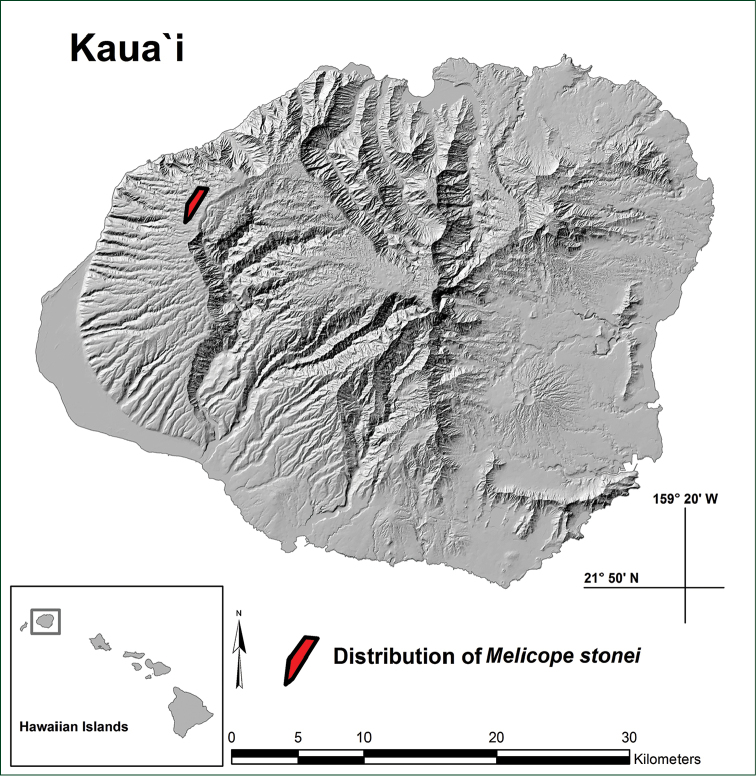
Map of Kaua‘i, Hawaiian Islands, with polygon indicating distribution of *Melicope stonei* in the Kōke‘e forests.

**Figure 4.**
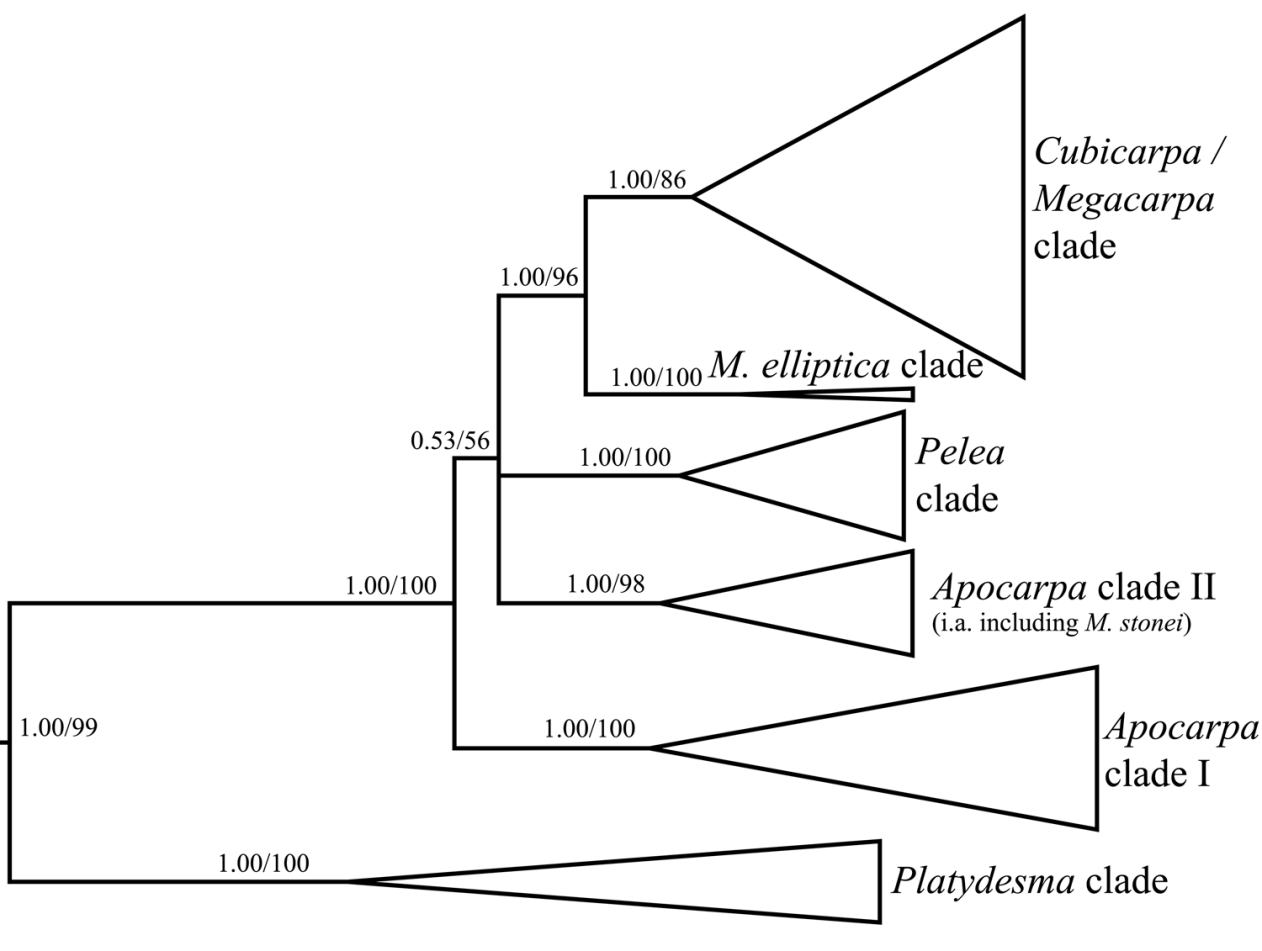
Phylogenetic placement of *Melicope stonei* K.R. Wood, Appelhans & W.L. Wagner based on four nuclear and two plastid markers (modified from Appelhans et al. 2014c). The phylogenetic tree only shows the Hawaiian radiation of *Melicope*. The terms *Apocarpa*, *Cubicarpa*, *Megacarpa* and *Pelea* refer to the former Hawaiian sections of *Melicope/Pelea* (Hartley & Stone 1989). The support values are displayed above the branches and the first value represents the Bayesian posterior probability values (pp), followed by the bootstrap values (bs) from the Maximum Likelihood analysis.

A striking character of *Melicope stonei* is the ramiflorous inflorescences, which it most notably shares with *M. clusiifolia*, *M. haleakalae*, *M. ovata*, *M. pseudoanisata*, *M. quadrangularis*, and *M. waialealae*, and can occur on rare occasions with other Hawaiian *Melicope*. This character is homoplasious and has evolved probably three or four times in Hawaiian *Melicope*: once in *M. stonei* and *M. ovata* of the *Apocarpa* group, once in the ancestor of *M. clusiifolia*, *M. haleakalae* and *M. waialealae*, who form the monophyletic *Pelea* group (Appelhans et al. 2014c; Fig. 4), and once or twice in the ancestor(s) of *M. pseudoanisata* and *M. quadrangularis*, which are part of an unresolved group of species belonging to the intermixed former sections *Cubicarpa* and *Megacarpa* (unpublished results; Fig. 4).

Morphologically, *Melicope stonei* is most similar to *M. barbigera* and the Kaua‘i population of *M. knudsenii* (see above). *Melicope knudsenii* has been shown to be polyphyletic and the Maui form does not belong to this group (Appelhans et al. 2014c; Fig. 4). As a result, a future publication is now in preparation which will resurrect the Maui form (i.e., *M. multiflora* Rock) as a species distinct from the Kaua`i *M. knudsenii*. While *M. barbigera* and the Kaua‘i form of *M. knudsenii* are resolved as immediate sisters in the phylogenetic analyses, *M. stonei* is not closely related to them and belongs to another clade of the paraphyletic *Apocarpa* group (Appelhans et al. 2014c; Fig. 4). It should be noted that the variable abaxial midrib pubescence of *M. stonei* ranging from villous on the sides to uniform pubescence (Fig. 2E–F) could represent natural variability or may suggest possible undetected hybridization between *M. stonei* and *M. barbigera*. Other putative *Apocarpa* hybrids collected and observed in the Kōke‘e forests of Kaua‘i include: *M. barbigera* × *M. haupuensis* (H. St. John) T.G. Harley & B.C. Stone (i.e., *Wood & Query 14696* [PTBG]) observed around Awa‘awapuhi and Honopū (825–1050 m); *M. haupuensis* × *M. pallida* (Hillebr.) T.G. Hartley & B.C. Stone (i.e., *Wood et al. 7725* [PTBG]) around Awa‘awapuhi (1050 m); and *M. ovata* × *M. stonei* (i.e., *Wood 17237* [BISH, K, MO, NY, PTBG, UC, US], *Wood 17245* [BISH, PTBG, UC, US], *Wood 17246* [BISH, PTBG, US]), in the valleys of Mākaha to Nu‘ololo (988–1097 m).

### Insert for existing key to Hawaiian *Melicope*

To accommodate *Melicope stonei*, the following couplets can be inserted into the existing key to Hawaiian *Melicope* (treated as *Pelea*) by Stone, Wagner, and Herbst (in Wagner et al. 1999, p. 1178) (Note: K=Kaua‘i; O=O‘ahu; WM=West Maui).

**Table:** 

1	Carpels distinct in fruit; leaves opposite or rarely ternate	**2**
1'	Carpels connate at least at base in fruit; the capsules 4-lobed; leaves opposite or whorled	**15**
2(1)	Endocarp pubescent, at least along suture	**3**
2'	Endocarp glabrous	**6**
6(2)	Leaves ternate; new growth black-resinous and minutely puberulent; exocarp glabrous and somewhat glaucous, especially when immature; K, O	***M. pallida***
6'	Leaves opposite; new growth not black-resinous, but puberulent or tomentose; exocarp glabrous or puberulent, but never glaucous	**7**
7(6)	Abaxial leaf surface densely long-villous or tomentose in a line along each side of midrib and sometimes on the midrib itself, the surface away from midrib densely to loosely pubescent or tomentose, some hairs tending to fall off with age	**7a**
7'	Abaxial leaf surface glabrous to densely tomentose or puberulent, sometimes more densely so toward midrib, but not long-villous in a line along each side of midrib	**8**
7a(7)	Midrib glabrous or nearly so other than long-villous hairs along sides; K	***M. barbigera***
7a'	Midrib pubescent and also long-villous or tomentose along sides; K	***M. stonei***
8(7)	Exocarp minutely puberulent throughout with short, erect, white hairs, becoming yellowish in dry specimens; O, WM	***M. elliptica***
8'	Exocarp glabrous or minutely appressed puberulent near suture	**9**
9(8)	Abaxial leaf surface densely tomentose or densely puberulent with strongly appressed, extremely fine hairs	**10**
9'	Abaxial leaf surface glabrous or densely puberulent when young, becoming sparsely so or glabrate with age	**11**
10(9)	Shrubs or trees to 3 m tall; leaves 1.5–5 cm wide; abaxial leaf surface densely puberulent with strongly appressed extremely fine hairs mixed with white waxy scurf; petioles 10–22 mm long; pedicels 5–10 mm long; O	***M. makahae***
10'	Large trees to 12 m tall; leaves (4–)6–11 cm wide; abaxial leaf surface densely tomentose without white waxy scurf; petioles 20–90 mm long; pedicels 1–5 mm long	**10a**
10a(10)	Leaf base weakly cordate; flowers (3–)20–200; cymes axillary, not ramiflorous; peduncles (10–)30–60 mm long; K, EM	***M. knudsenii***
10a'	Leaf base rounded; flowers 3–5(–7); cymes axillary and ramiflorous; peduncles 1–5(–10) mm long; K	***M. stonei***

## Supplementary Material

XML Treatment for Melicope stonei
